# Effect of Drying Methods on the Phenolic Profile and Antioxidant Capacity of *Pithecellobium dulce* (Roxb.) Benth. Aril and Its Inhibitory Properties on Human SW480 Colon Adenocarcinoma Cells

**DOI:** 10.3390/molecules30020233

**Published:** 2025-01-09

**Authors:** Ángel Félix Vargas-Madriz, Aarón Kuri-García, Ivan Luzardo-Ocampo, Roberto Augusto Ferriz-Martínez, Teresa García-Gasca, Carlos Saldaña, Haidel Vargas-Madriz, Salvador Horacio Guzmán-Maldonado, Jorge Luis Chávez-Servín

**Affiliations:** 1 Laboratorio de Biología Celular y Molecular, School of Natural Sciences, Universidad Autonoma de Queretaro, Av. de las Ciencias S/N, Juriquilla, Queretaro 76230, Mexico; angel.vargas@uaq.mx (Á.F.V.-M.); aaron.kuri@uaq.mx (A.K.-G.); roberto.augusto.ferriz@uaq.mx (R.A.F.-M.); tggasca@uaq.edu.mx (T.G.-G.); 2 Institute for Obesity Research, Tecnológico de Monterrey, Av. Eugenio Garza Sada 2501, Monterrey 64841, Mexico; ivanluzardo@tec.mx; 3 School of Engineering and Sciences, Tecnologico de Monterrey, Av. General Ramón Corona 2514, Zapopan 45138, Mexico; 4 Laboratorio de Biofísica de Membranas y Nanotecnología and Laboratorio Nacional de Visualización Científica Avanzada (LAVIS), School of Natural Sciences, Universidad Autonoma de Queretaro, Av. de las Ciencias S/N, Juriquilla, Queretaro 76230, Mexico; carlos.saldana@uaq.mx; 5 Departamento de Producción Agrícola, Centro Universitario de la Costa Sur, Universidad de Guadalajara—UDG, Av. Independencia Nacional 141, Guadalajara 48900, Mexico; haidel_vargas@hotmail.com; 6 Laboratorio de Alimentos, Centro de Investigación Regional del Centro, Instituto Nacional de Investigaciones Forestales, Agrícolas y Pecuarias (INIFAP), Campos Experimental Bajío, Km. 6, Carr. Celaya-San Miguel de Allende, Celaya 38810, Mexico; shoraciogm@gmail.com

**Keywords:** *Pithecellobium dulce*, anti-cancer, antioxidant, drying methods, phenolic compounds

## Abstract

*Pithecellobium dulce* (*P. dulce*) is a Mexican plant that is consumed raw or in different preparations, and its anti-inflammatory and antioxidant properties have traditionally been useful in treating several conditions. However, the post-harvest drying process can alter the content of bioactive compounds in *P. dulce*. This study aims to evaluate the impact of different drying methods on the phenolic profile and antioxidant capacity of this plant, as well as its inhibitory effect on human SW480 colon adenocarcinoma cells. After oven drying, the samples showed a higher amount (*p* < 0.05) of phenolic compounds, up to 1149.45 ± 69.27 mg GAE/100 g LE, which is 80% more than the freeze-dried samples. Also, the antioxidant capacity was higher in oven-dried samples, with 44.63 ± 2.00 µmol Trolox equivalents/g LE, 108% more than the freeze-dried method. Methanolic extraction, in turn, yielded better results than aqueous and ethanolic extractions. Up to 14 polyphenolic compounds were detected in oven-dried samples. For in vitro assays in SW480 cells, the 50% *v*/*v* methanolic extract was used. From this extract, the median lethal concentration (LC_50_) was determined to be 13.76 mg/mL, which represents the concentration necessary to inhibit the growth of half of the cancer cells of this cell line. The extract led to cell cycle arrest in the G1 phase and an increase in apoptosis-induced cell death. The *P. dulce* extract augmented p53 and decreased KRAS gene expressions. Results suggested pro-apoptotic mechanisms in colon cancer cells in vitro linked to *P. dulce* bioactive compounds, which are better preserved when oven-dried plants are subjected to methanolic extraction.

## 1. Introduction

Beyond the nutritional properties of food products of vegetable origin, the bioactive compounds they contain have been linked to a wide range of health benefits, and their use for possible therapeutic purposes has been suggested. Among these, polyphenols have demonstrated beneficial effects against several chronic non-communicable diseases, such as cancer, most of which are attributable to their antioxidant and anti-inflammatory properties [1].

Currently, cancer is one of the most prevalent non-communicable diseases worldwide, and it is the second leading cause of death. Overall, cancer can be defined as an irreversible impairment of cellular homeostasis due to intrinsic factors (mutations and genetic or metabolic alterations) or extrinsic ones (UV exposure, tobacco, stress, among others) [2,3]. Colorectal cancer (CRC) is one of the most frequently diagnosed cancers today, and its prevalence has been rising in the last few years, and it is expected to grow by more than 60% by 2030. CRC is linked primarily to sedentary lifestyles, high consumption of calorie-rich and processed products, and low intake of vegetable-origin food products [4]. It is important to note that there is no current scientific consensus on the definition of what is called “ultra-processed” foods, nor on how the degree of processing or the presence of an excess of certain ingredients inherent to these foods produce adverse health effects [5].

Polyphenols found in several plants have drawn the attention of the food and pharmaceutical industries due to their functional properties in the prevention and treatment of non-communicable diseases. Furthermore, the manner in which these plants are collected and stored and how the polyphenols are extracted are also of industrial interest [6,7]. Selection of the correct post-harvest methods is important in maintaining the chemical composition of food products, while the optimum drying process allows their preservation by inhibiting the growth of microorganisms and inactivating degradation enzymes [8,9,10]. Some research works have reported that freeze-drying is an optimal method for maintaining the physicochemical properties of vegetable samples, but special equipment and extensive drying times are needed, and such equipment has a limited capacity for handling large sample volumes [6]. Hot air-drying, on the other hand, can degrade and oxidize bioactive compounds from vegetable samples, but controlled heat at 40–80 °C promotes a higher release of phenolic compounds than does freeze-drying, although this depends on additional factors, such as the food matrix, extraction process, and solvents used, among others [11].

*Pithecellobium dulce* (Roxb.) Benth. (*P. dulce*) is a tree native to the Americas, which is widely distributed in Mexico, mainly in tropical zones. *P. dulce* belongs to the Leguminosae family and the Mimosoideae subfamily, and it is called “Guamúchil” in Mexico and “Ingraji chinch” or “Manila Tamarind” in India [6,12]. In Mexico, between the months of February and August, it produces a variegated green and red coiled pod, which contains 5 to 12 white arils with black seeds inside [6]; the aril is the fleshy part that surrounds the seed. Several studies have demonstrated its anti-inflammatory, antioxidant, antibacterial, hypoglycemiant, and antiproliferative properties, among others [13]. For instance, *P. dulce* aril methanolic extract inhibited pulmonary metastasis in vivo by reducing the expression of pro-inflammatory genes while also modulating cell cycle-related genes [14]. Some phenolic compounds in the aril of *P. dulce* have been observed to have anticancer effects; for example, epigallocatechin 3-gallate (EGCG) has been shown to cause apoptotic cell death with the release of cytochrome c in CRC cells, in addition to inhibiting KRAS-induced cell proliferation in epithelial cells and blocking the G1 phase [15]. Furthermore, it has been observed in colon cancer cell lines (SW480, HCT116, HT29, Caco-2) that caffeic acid, p-Coumaric acid, and gallic acid increase the activity of the G0/G1 cell phase, decrease the S cell phase and increase cell death by apoptosis [16]. Cell proliferation involves the aforementioned phases: the G0/G1 phase corresponds to a state of quiescence (G0), and cells are subsequently stimulated to enter the first cell growth cycle (G1) and prepare for DNA replication. The DNA replication phase occurs in the S phase, and then the cells enter the G2 phase, which is a growth process to correct errors in DNA duplication before entering the mitosis phase (M), in which there is cell division [17]. The background information on the *P. dulce* plant suggests that bioactive compounds in the aril may play an inhibitory role against CRC cells. To date, there is no article discussing the effect of the drying method on the phenolic profile and antioxidant capacity of *P. dulce* aril or its anticancer effect on colon cancer cell lines. For this reason, this article aims to evaluate the impact of two drying methods (oven drying and freeze-drying) on the polyphenolic profile and antioxidant capacity of the *P. dulce* aril and its inhibitory properties on SW480 human colon adenocarcinoma cells. The results of this research could provide useful information for optimizing polyphenolic compounds through the drying process and use of solvents, in addition to providing information on the biological effect of *P. dulce* extracts on CRC cells.

## 2. Results

### 2.1. Impact of Drying Methods on the Polyphenolic Composition and Antioxidant Capacity of P. dulce Aril

As observed in Table 1, total phenolic compounds (hereinafter, TPC) ranged from 631.72 to 1149.45 mg GAE/100 g LE, total flavonoids (TF) ranged from 5.10 to 20.93 mg CE/100 g LE, and condensed tannins (CT) presented values between 0.07 and 0.19 mg CE/100 g. Except for CT, the oven-dried samples displayed higher (*p* < 0.05) TPC and TF than the freeze-dried samples. Overall, aqueous extracts of oven-dried samples showed the lowest amount of TPC and TF, while hydroalcoholic extracts of the freeze-dried samples showed higher amounts of TPC, TF, and CT than their aqueous counterparts.

The range of values for antioxidant capacity (Table 2) were 19.10–44.63, 27.93–35.03, and 143.81–202.23 µmol Trolox equivalents/g LE for DPPH, FRAP, and ABTS, respectively. Oven-dried samples displayed a higher antioxidant capacity than freeze-dried samples, while hydroalcoholic extracts showed higher values than aqueous extracts.

A total of 14 compounds were identified and quantified in *P. dulce* aril using HPLC-DAD, including 3 hydroxybenzoic acids (ellagic, 4-hydroxybenzoic, and gallic acids), 4 hydroxycinnamic acids (sinapic, chlorogenic, caffeic, *p*-coumaric, and ferulic acids), and 4-hydroxyphenylacetic acid for the phenolic acids (Table 3).

**Figure 1:**
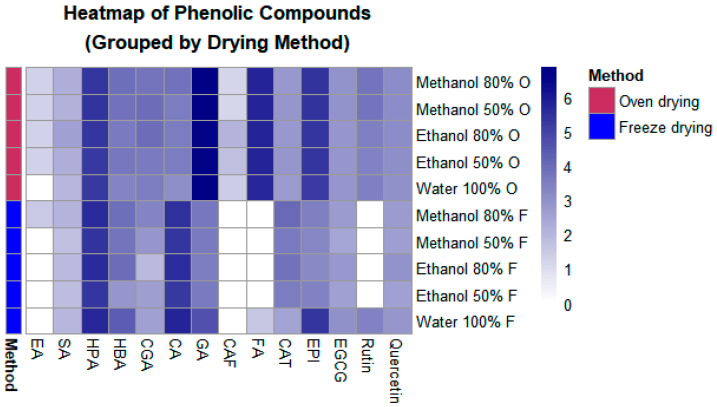
Relative *P. dulce* aril phenolic compounds concentration by drying types (oven and freeze-drying) and solvent concentrations (ethanol, methanol, and water). The relative concentration corresponds to the logarithmically transformed values (log1p) of the concentrations of the phenolic compounds obtained by HPLC-DAD. CAF: Caffeic acid; CAT: (+)-Catechin; CGA; Chlorogenic acid; CA: *p*-Coumaric acid; GA: Gallic acid; EA: ellagic acid; EPI: (−)-Epicatechin; EGCG: (−)-Epigallocatechin-3-*O*-gallate; FA: Ferulic acid; HPA: Hydroxyphenylacetic acid; HBA: Hydroxybenzoic acid; SA: Sinapic acid.

Regarding flavonoids, 3 flavanols [(+)-catechin, (−)-epicatechin, and (−)-epigallocatechin-3-*O*-gallate] and 2 flavonols (rutin and quercetin) were identified. To simplify the grouping, a heatmap was drawn up (Figure 1). Among the phenolic acids, gallic and ferulic acids exhibited the highest concentrations (Table 3). In the case of freeze-dried samples, 13 compounds were detected; however, some compounds, such as ferulic acid, were detected only in aqueous extracts from freeze-dried samples. Freeze-dried samples exhibited a higher amount of *p*-coumaric acid than oven-dried samples, particularly in the aqueous extract.

Among the flavonoids, (+)-catechin showed higher amounts in the hydroalcoholic extracts from freeze-dried samples than from oven-dried samples; (−)-epicatechin was the highest in the oven-dried samples, and a similar trend was observed for (−)-epigallocatechin-3-*O*-gallate and quercetin (Table 4). Overall, M:W extractions yielded the highest amounts of flavonoids.

Phenolic acids and flavonoids in the oven-dried samples were higher than in freeze-dried samples (Figure 1). Amounts of hydroxyphenylacetic and gallic acids were notably higher, regardless of the drying or extraction method. It is observed that M:W extracts contained all compounds and displayed some of the highest amounts, which indicates that this may be the optimum method.

**Figure 2:**
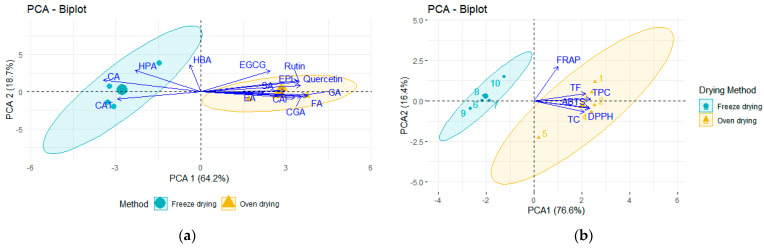
Principal component analysis (PCA) of *P. dulce* aril measurements (phenolic compounds and antioxidant capacity) obtained from oven-drying and freeze-drying samples. (**a**) Phenolic acids; (**b**) Spectrophotometric measurements. ABTS: 2,2-Azino-bis(3-ethylbenzothiazoline-6-sulfonic acid); CAF: Caffeic acid; CAT: (+)-Catechin; CGA; Chlorogenic acid; CA: *p*-Coumaric acid; GA: Gallic acid; DPPH: 1,1-Diphenyl-2-picrylhydrazil assay; EA: ellagic acid; EPI: (−)-Epicatechin; EGCG: (−)-Epigallocatechin-3-*O*-gallate; FA: Ferulic acid; FRAP: Ferric reducing antioxidant power; HPA: Hydroxyphenylacetic acid; HBA: Hydroxybenzoic acid; SA: Sinapic acid.

Figure 2 shows a PCA analysis of the phenolic compounds identified in *P. dulce* extractions using HPLC-DAD. A clear difference between the two drying methods was observed for phenolic acids (Figure 2a) and spectrophotometric analyses (Figure 2b). The hydroalcoholic extracts (80% *v*/*v*) displayed the highest extraction of phenolic acids but varied depending on the drying method applied to the original sample. The oven-dried samples were more closely related to ferulic acid, chlorogenic acid, quercetin, and rutin. In contrast, the freeze-dried samples were more closely related to *p*-coumaric and hydroxyphenylacetic acids. The principal components explored accounted for 82.9% of the total variation (Supplementary Tables S2 and S3).

### 2.2. Effect of P. dulce Extracts on Cellular Metabolic Activity, Apoptosis, Cell Cycle, Necrosis, and the Expression of Pro-Apoptotic and Anti-Apoptotic Genes

**Figure 3:**
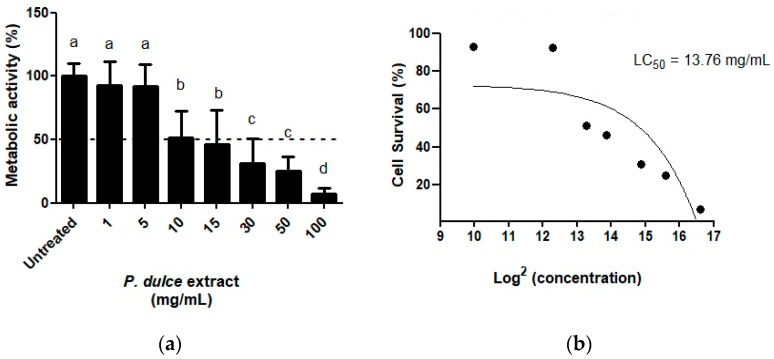
Cytotoxicity assessment of *P. dulce* aril extracts on human SW480 colon adenocarcinoma cells. (**a**) Impact of *P. dulce* extracts on the cellular metabolic activity. The results are expressed as the mean ± SD of three independent experiments in triplicate. Different letters represent significant differences (*p* < 0.05) according to Tukey–Kramer’s test. The dashed line in (**a**) indicates a metabolic activity of 80%. Untreated cells were used as a negative control (DMEM + 0.5% bovine serum albumin, BSA). Triton X-100 was used as a positive control. (**b**) Adjusted dose-response and calculation of the half-lethal concentration (LC_50_). The black dots represent the survival percentage depending on the concentration of *P. dulce* aril extracts used.

As shown in Figure 3, *P. dulce* concentrations higher than 10 mg/mL displayed cytotoxic effects (Figure 3a) by reducing cellular metabolic activity. Calculations of the half-lethal dose concentration (LC_50_) using a dose-response adjusted curve (Figure 3b) showed that a concentration of 13.766 mg/mL is needed to achieve half of the cells’ death. Based on the composition of *P. dulce* extracts, LC_50_ was equivalent to 0.35 ± 0.01 µg eq. ellagic acid/100 g LE, 5.36 ± 0.06 µg eq. 4-hydroxybenzoic acid/100 g LE, 112.63 ± 6.28 µg eq. gallic acid/100 g LE, 1.14 ± 0.04 µg eq. sinapic acid/100 g LE, 5.05 ± 0.09 µg eq. chlorogenic acid/100 g LE, 0.63 ± 0.02 µg eq. caffeic acid/100 g LE, 4.51 ± 0.14 µg eq. *p*-coumaric acid/100 g LE, 41.39 µg eq. ferulic acid/100 g LE, 26.69 ± 0.32 µg eq. 4-hydroxyphenylacetic acid, 2.17 ± 0.02 µg eq. (+)-catechin/100 g LE, 27.39 ± 0.11 µg eq. (−)-epicatechin, 2.18 µg eq. (−)-epigallocatechin-3-*O*-gallate/100 g LE, 4.07 µg eq. rutin/100 g LE, and 2.84 µg eq. quercetin/100 g LE.

**Figure 4:**
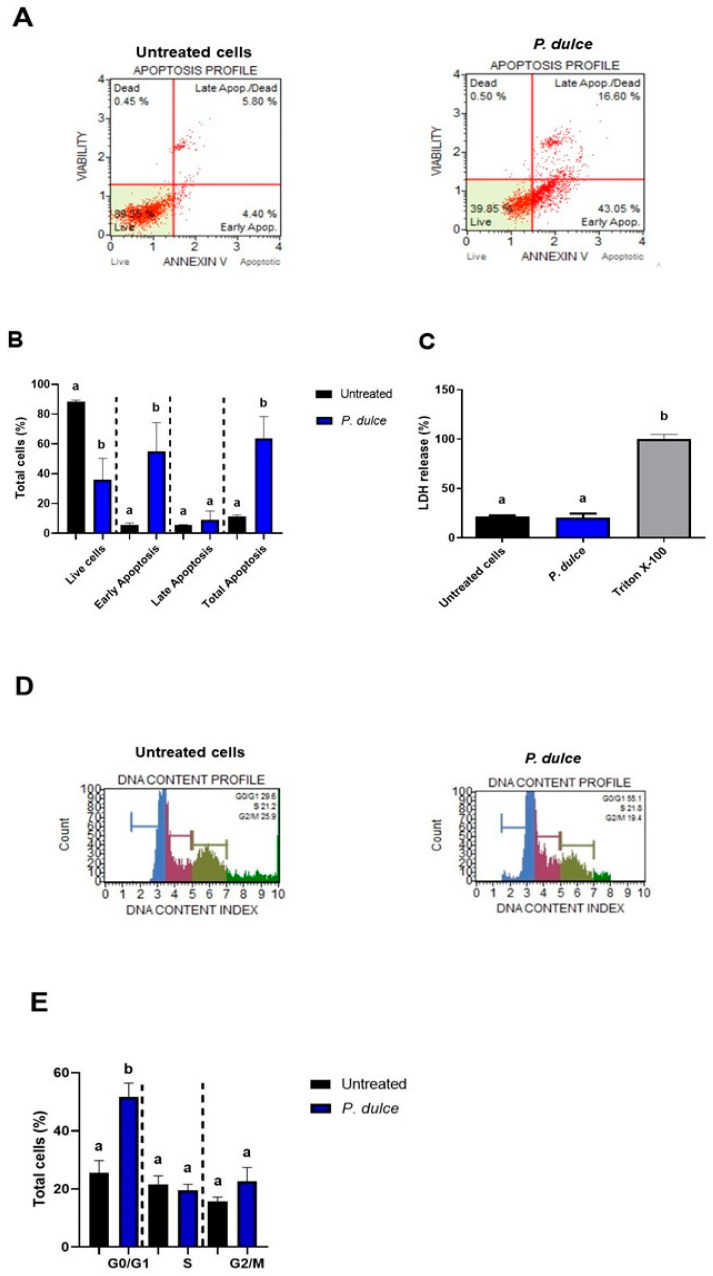
Effect of *P. dulce* aril extracts on apoptosis, cell cycle, and necrosis (LDH assay) of human SW480 colon adenocarcinoma cells. (**A**) Representative images of SW480 cell apoptosis after LC_50_ treatment of *P. dulce,* analyzed by flow cytometry; (**B**) quantification of the total (%) of living cells, cell death by early apoptosis, late apoptotic and total apoptotic cells; (**C**) release of lactate dehydrogenase (LDH) after treatment with *P. dulce*; (**D**) representative images of the effect on the cell cycle after *P. dulce* challenging. Blue color G0/G1, magenta color S, opaque green color G2/M; (**E**) quantification (%) of living cells during each phase of the cell cycle. Different letters express significant differences (*p* < 0.05) according to Tukey–Kramer’s test. For (**B**,**D**), a statistical comparison was performed between treatments for each cell death classification. For (**C**), the statistical comparison was performed among all groups. The negative control corresponded to untreated cells (DMEM + 0.5% BSA). For the LDH assay, Triton X-100 was used as a positive control. Triton X-100 (TX) was used as a positive control in the cytotoxicity assay, demonstrating 100% toxicity. Triton X-100 is a nonionic detergent that causes cell lysis by disrupting plasma membranes, which allowed the study to arrive at a reference value for evaluating the efficacy of treatments with *P. dulce* extracts. The high toxicity observed in Triton X-100 confirms its ability to induce complete cell death, providing a comparative measure for the cytotoxic effects of the tested extracts [18].

Two types of cell death were evaluated in human SW480 cells after their exposure to LC_50_ of *P. dulce* (Figure 4). Representative images of live, dead, early apoptotic, and late apoptotic cells are presented in Figure 4A, where their quantification (Figure 4B) showed the highest amounts of live cells in the negative control, as expected, and most of *P. dulce*-treated cells in early and late apoptosis. Regarding necrosis (Figure 4C), indirectly measured by lactate dehydrogenase (LDH) release, no differences were shown between untreated and *P. dulce* LC_50_-treated cells, suggesting the absence of necrotic mechanisms in this model, although additional necrosis-related parameters should be assessed to conclude fully about this cell death type. Representative histograms of cell populations in each cell cycle stage (Figure 4D) and their quantification (Figure 4E) indicated that most of the cells affected by *P. dulce* treatments are located at the G0/G1 stage, followed by G2/M and S phases.

**Figure 5:**
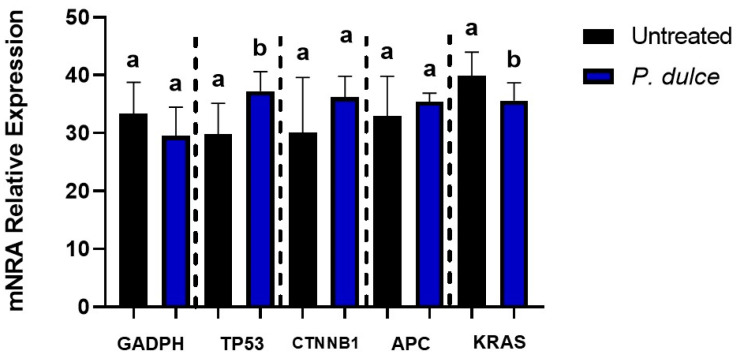
Impact of *P. dulce* LC_50_ concentration on the relative mRNA expression of pro-apoptotic and anti-apoptotic genes. The results are expressed as the mean ± SD of three independent experiments in triplicate. Different letters express significant differences (*p* < 0.05) according to Tukey–Kramer’s test. Gene expression was calculated using the 2^−ΔΔCt^ method.

Figure 5 showed differences between untreated and *P. dulce*-treated SW480 cells for just the Tp53 and KRAS expression, where mRNA expression was lower (*p* < 0.05) than in untreated SW480 cells.

## 3. Discussion

The aim of this research was to assess the impact of the drying method on the phenolic profile (phenolic acids and flavonoids) and antioxidant capacity of *P. dulce* aril extracts (aqueous and hydroalcoholic) and to evaluate the inhibitory and pro-apoptotic effects of the best extract on human SW480 colon adenocarcinoma cells. The results indicated that freeze-dried samples presented fewer polyphenols and lower antioxidant capacity than oven-dried samples. The formation of ice crystals within the plant matrix has been suggested, which may keep the cellular structure intact, thus retaining the high molecular weight of conjugated bioactive compounds of the plant sample during freeze-drying. On the contrary, oven drying may induce an increase in the rupture of the cell wall of the plant sample [19]. The results obtained are similar to those reported by Martínez García et al. [20] in leaves of *Urtica dioica* L., observing that drying at a temperature of 35 °C was more effective than freeze-drying for extracting phenolic compounds from the plant sample. Our results regarding the superiority of oven-dried samples can be explained by the heat-induced separation of polyphenols originally bound to the plant matrix and subcellular compartments, producing phenolic aglycones. By inducing heat, antioxidants (phenolic compounds) bound to the cell wall may be released, accompanied by the heat inactivation of degrading enzymes such as polyphenol oxidase. In addition, new antioxidants may be generated as products of the Maillard reaction (melanoidins) resulting from the thermal chemical reaction [20]. The Maillard reaction is a complex non-enzymatic browning reaction that occurs between reducing sugars and amino acids in the presence of heat, forming melanoidins with antioxidant properties. It has been observed, for example, that when roasting coffee, chlorogenic acids are incorporated into the melanoidins, mainly in condensed form, while a minor portion remains bound to esters. Some of the chlorogenic acids remain intact, and their degree of incorporation depends on the initial content [21].

Although freeze-drying has been highlighted as one of the best methods to preserve polyphenols, dried vegetable samples (30–120 °C) have presented a higher amount of bioactive compounds than freeze-dried samples [22]. Extensive investigation is therefore necessary into the unique interactions between bioactive compounds and the food matrix, as different biological structures and physicochemical characteristics definitively affect the yield of bioactive compounds [23]. In addition, further research is needed into the best solvents for extracting the compounds, as there is no one universal solvent that offers optimum polyphenol extraction [24,25]. Previous research has assayed ethanol, methanol, water, and several mixtures to extract phenolics without any clear trend as to which is the best solvent for this purpose; clearly, however, aqueous extracts have been found to be less pure because the water also extracts sugars and soluble proteins, among other compounds [6].

The values reported in this research for TPC in aqueous extracts coincide with those of other reports [26,27]. In hydroalcoholic extracts, TPC values are lower than those reported by Rao et al. [28] but higher than those reported by other authors [29,30,31]. On the other hand, TFC values are lower than those reported for hydroalcoholic extracts, which may be attributable to agroclimatic factors, fruit maturation, and harvesting location [6]. *P. dulce* contains lower TFC than other wild plants, such as *Psidium guajava* Linn or *Pouteria campechiana* [30]. The amounts of compounds identified using HPLC-DAD concur with other reports for *P. dulce* [6], but the drying process significantly affects the amount of each identified polyphenol. For instance, freeze-drying may result in a lower content of certain polyphenols than oven-drying procedures [32]. However, contrary to the assumption of degradation during freeze-drying, it is more plausible that conjugated and high molecular weight derivatives remain preserved in their native form during the process. The absence of ellagic acid and hydroxycinnamic acids in the freeze-dried samples would support this hypothesis. In the freeze-drying process, it has been observed that slow freezing can cause cell wall rupture by the formation of large ice crystals that diffuse outwards [33]. In our study, rapid freezing probably occurred, preserving the ice crystals within the cell wall and thus keeping the phenolic compounds intact. This effect may also be favored by the type of plant tissue, as reported by Nowak et al. [33], who highlighted the influence of the integrity of the internal tissue of the plant material on the efficiency of freeze-drying [34]. Oven drying, on the other hand, facilitates thermal hydrolysis of the conjugates, leading to the presence of phenolic compounds such as caffeic and ferulic acid in their free forms. In addition, new antioxidant compounds are likely to be generated, probably due to the availability of phenolic precursors by non-enzymatic interconversion between molecules of phenolic compounds [35], as previously mentioned.

Various studies have shown that bioactive compounds derived from plants may be useful in preventing or treating different metabolic diseases, including cancer, demonstrating anti-inflammatory effects, regulation of cell proliferation, and protection against DNA oxidative damage [36]. There are valuable opportunities for research into natural products containing bioactive compounds with anticancer effects and fewer side effects than current treatments, within which plant-based foods and their extracts are a viable option [37].

In the present study, the *P. dulce* extract demonstrated antiproliferative effects with an LC_50_ of 13.76 mg/mL. This value can be considered high compared to previous studies where IC_50_ concentrations are handled in µg/mL. Dhanisha et al. [14] observed cytotoxic effects using a 50% methanol/water extract of *P. dulce* aril in A549 and B16F10 cell lines (alveolar basal epithelial adenocarcinoma). Their results showed IC_50_ values of 119 and 114 μg/mL, lower concentrations than those reported in the present study. In another study using the same extract, these authors observed cytotoxicity in DLA cells (Dalton’s Lymphoma Ascites cells) with IC_50_ values of 50, 100, and 250 μg/mL [14]. Other studies have reported the cytotoxic and antiproliferative effects of different parts of the *P. dulce* tree. For example, Alhamed et al. [38] determined the cytotoxic effects of *P. dulce* seed using an 80% *v*/*v* methanolic extract on the Lovo colon cancer cell line (epithelial cells). Their study found that 3 μg/mL of the seed extract reduced Lovo cell viability by 50% (IC_50_). On the other hand, Sharma [39] used an aqueous extract of *P. dulce* leaves in MCF-7 cells (human breast carcinoma), demonstrating a dose-dependent cytotoxic effect with an IC_50_ concentration of 400 μg/mL. These studies indicate that the antiproliferative effect of *P. dulce* extracts varies across different cell lines, which may be due to the susceptibility of the cell lines to the bioactive compounds in *P. dulce* extracts [40]. On the other hand, the LC_50_ obtained in this research does not invalidate the observed effectiveness; the variability in the metrics could be explained by the different objectives evaluated in the studies (inhibition vs. lethality), the experimental methods, the concentration of bioactive compounds present in the extract, or even the specific characteristics of the cell lines used [40,41]. The difference between IC_50_ and LC_50_ is crucial to interpret the results of this study. In the case of the IC_50_, a low value is considered to have high toxicity, while a high LC_50_ value, such as the one reported in this study, suggests lower toxicity, which may be an advantage for therapeutic applications in which a more selective effect on cancer cells is sought and potentially minimize adverse effects on healthy cells [41]. Furthermore, cytotoxicity alone cannot be taken as evidence of an antineoplastic effect; apoptotic effects in these cell lines must also be demonstrated [39].

The observed inhibition of cell growth in SW480 cells caused by the cytotoxic effects of *P. dulce* aril extract may be due to one or more bioactive components of this plant. In addition to the cytotoxic effects, understanding the type of cell death that the *P. dulce* extract may induce and the mechanisms leading to this type of cell death is important. As is well known, apoptosis is a controlled cell death mechanism that prevents uncontrolled cell proliferation [37]. During colon carcinogenesis, transformed cells proliferate uncontrollably, so extracts or compounds capable of activating cell death via apoptosis are considered anticancer agents in colon cancer therapy [37]. In our study, the LC_50_ concentrations of the methanolic extract of *P. dulce* resulted in significantly more cells undergoing total apoptosis than the control. These results are similar to those reported by Dhanisha et al. [42], where the methanolic extract of *P. dulce* aril caused damage at the cellular membrane level in DLA cells, leading to DNA fragmentation and apoptosis.

In this study, the authors analyzed the effects of *P. dulce* extract on cell cycle phases, showing an increase in the G0/G1 phase. In mammals, healthy cells are controlled by various protein complexes formed by cyclins and cyclin-dependent kinases. Healthy cells must go through the G1, S, G2, and M phases through a controlled mechanism to generate two new cells. However, in the case of colorectal cancer (CRC), malignant cells proliferate without this control [43]. It has been observed that some phenolic compounds can intervene in regulating the cell cycle in CRC cells. For example, it has been demonstrated that caffeic acid has anticancer properties in SW480 cells, arresting the cell cycle at the G0/G1 phase [16]. Additionally, caffeic acid has been shown to have antiproliferative effects in HT-29 and HCT-15 colon cancer cells, with the proposed effect possibly mediated by phenolic compounds present, which cause cell cycle arrest in the sub-G1 phase, as well as damage at the cellular membrane level through ROS generation, thus activating p53, upregulating Bax, and downregulating Blc2 [44]. In another study, gallic acid demonstrated antiproliferative effects in SW480 cells, and its effects were analyzed at different phases of the cell cycle, causing cell cycle arrest in the S and G2/M phases, possibly due to alterations in DNA replication [45]. Furthermore, epigallocatechin has been shown to inhibit the activity of *p27* and *p21* cyclin-dependent kinases [43]. Another phenolic compound identified in *P. dulce* aril, rutin, has been shown to have cytotoxic effects in SW480 cells and to improve metabolism in these cells by arresting the cell cycle at the G1 phase, leading to apoptosis [46].

In the present study, a significant increase in the expression of Tp53 and a decrease in the expression of KRAS, two antagonistic genes that perform the functions of apoptosis and cell proliferation, respectively, were observed [47]. Tp53 is a tumor suppressor gene (encoding the p53 protein) that is in an active state and is related to apoptosis and cell cycle arrest when DNA damage is detected [48]. The target gene p21 is a member of the cyclin-dependent kinase inhibitors (cell cycle regulators). It is located downstream of p53 and participates in the cellular arrest of the G1, S, and G2/M phases induced by p53 and other genes [49]. During CRC, the p53 protein is usually suppressed, which leads to a reduction in the transcription of genes that promote apoptosis, allowing uncontrolled proliferation of cancer cells [40,48,50]. In our study, the increase in Tp53 expression may be due to the bioactive compounds present in the *P. dulce* extract. This suggests that phenolic compounds could be reactivating the function of p53, promoting cell cycle arrest in the G1 phase and inducing programmed cell death [15]. Other studies have observed that some phenolic compounds such as quercetin, ferulic acid, caffeic acid, (−)-Epigallocatechin-3-*O*-gallate; found in the aril of *P. dulce*, positively regulate the p53, p21, and p27 proteins. This may occur by increased acetylation or phosphorylation of p53, which causes G0/G1 cell cycle arrest, improves its stabilization and binding to DNA, and induces apoptosis; this in different types of cancer cell lines such as ME180, CRPC, HaCaT [15,51]. Our results coincide with those reported by Dhanisha et al. [8], who also found increased expression of p53 in DLA cells after treatment with a methanolic extract of *P. dulce*. These authors also observed an increase in the expression of other apoptotic enzymes, such as caspase 3, caspase 9, and Bax, all important for the execution of cell death by apoptosis. The above indicates that the *P. dulce* extract exerts an anticancer effect.

The APC gene is another tumor suppressor protein that is mutated in CRC. Mutation of this protein prevents β-catenin degradation, thereby activating the Wnt pathway, which inhibits enzymes involved in apoptosis, such as caspase 3, caspase 9, and cytochrome c, allowing malignant cells to evade apoptosis and leading to CRC progression [52]. However, in the present study, no significant differences were found in APC and β-catenin expression.

KRAS, for its part, is an oncogene that regulates cell growth and differentiation. In an inactive state, it can be bound to GDP (guanosine diphosphate), and in an active state, it can be bound to GTP (guanosine triphosphate). Under normal conditions, both states alternate [53]. However, KRAS is frequently mutated in CRC, causing the protein to remain in the active state without being able to be inactivated. This leads to the activation of KRAS downstream signaling pathways that promote cell proliferation and survival, such as the MAPK/ERK pathway and the PI3K/AKT pathway [41,48,54]. Consequently, cell growth, proliferation, and survival with resistance to apoptosis occur. In the present study, this oncogene presents a significant decrease in its expression after treatment with *P. dulce* extract. This decrease could be related to a reduction in the activation of ERK and AKT, which causes cell cycle arrest and death by apoptosis [51]. This suggests that *P. dulce* extract may have an impact on the regulation of signals that promote CRC progression. The decrease in KRAS in the present study could be due to the presence of some phenolic compounds found in *P. dulce* extract, such as quercetin, since this flavonoid has been shown to reduce KRAS expression, particularly in cell lines with mutations in this oncogene, as is the case of SW480 cells [54,55]. In other studies, epicatechin has been observed to act as a potent modulator of the MAPK/ERK pathway and the PI3K/AKT pathway by inhibiting the activation of these pathways during oncological mutations [15]. In addition, Maugeri et al. [55] mentioned that quercetin induces apoptosis in DLD-1, COLO205, HT29, and WIDR cell lines by inhibiting AKT and activating JNK. This latter protein contributes to the upregulation of pro-apoptotic proteins such as Bax and caspase-3 and the downregulation of Bcl-2, favoring apoptosis [15]. Rutin, another flavonoid present in the extract, plays a key role in modulating intracellular signaling pathways (cell proliferation and death). This flavonoid has been shown to significantly influence MAPK signaling by reducing death receptors 4 and 5 (DR4/DR5), as well as AKT, ERK, and NF-κB proteins, both in vitro and in vivo studies [41]. EGCG has also been shown to inhibit KRAS -induced cell proliferation in intestinal epithelial cells, in addition to blocking cell cycle transition in the G1 phase through the inhibition of cyclin D1 expression [15]. This suggests that the bioactive compounds in the *P. dulce* extract may decrease signals that favor cell proliferation and survival in cancer cells.

Overall, the results suggest that the methanolic extract of *P. dulce* not only exerts a cytotoxic effect but, due to its reported phytochemical compounds, activates programmed cell death mechanisms to stop cell proliferation in colon cancer, thus exhibiting anticancer and pro-apoptotic effects. This could be due to the regulation of oncogenes and tumor suppressor genes such as KRAS and Tp53, as well as the modulation of the aforementioned signaling pathways. However, the individual components must be thoroughly analyzed in order to fully understand their effects on CRC, and in vivo studies must be conducted with this plant material.

## 4. Materials and Methods

### 4.1. Plant Material

*Pithecellobium dulce* (Roxb.) Benth. aril was registered in the World Flora database (https://worldfloraonline.org/taxon/wfo-0000178252, accessed on 8 March 2023). Aril collection was conducted in Jalpa de Serra (Queretaro, Mexico) during the spring-summer 2021 season. The sample was identified and registered by a specialist with the Jerzy Rzedowski Herbarium of the Universidad Autonoma de Queretaro. Samples were cleaned and subjected to one of two drying procedures: (1) oven drying (Shel-Lab Fx 1375, Swedesboro, NJ, USA) at 40 °C for 48 h and (2) freeze-drying at −55 °C and 1 Pa in a freeze-dryer (10 N Series, SCIENTZ, Ningbo, Zhejiang, China). Once dried, the samples were ground using an electric mill and screened through a 0.5 mm sieve. The resulting samples were stored in sealed bags at −80 °C for further processing.

### 4.2. Extraction Process

The methodology used for the extraction was based on Godínez-Santillán et al. [56] with slight modifications. The dry powder sample was used to prepare various extracts by mixing 5 g of sample with 500 mL of solvent with a solid: liquid ratio (1:100 g/mL). The solvents used were ethanol–water (80:20 and 50:50 *v*/*v* E:W), methanol–water (80:20 and 50:50 *v*/*v* M:W), and 100% water. Distilled water, ethanol, and HPLC-grade methanol were used for the preparations. Magnetic stirring (model OM10E, brand OVAN, Burladingen, Germany) was performed at 100 rpm and at 22 ± 1 °C for 16 h without light. The solutions were then filtered through Whatman 541 paper (Cytiva, 110 mm diameter, hardened and ash-free, purchased from Sigma-Aldrich, St. Louis, MO, USA) and concentrated in a rotary evaporator (model R-200, Büchi, Flawil, Switzerland) at 40 °C under a vacuum pressure of 100 mmHg. Finally, the extracts were lyophilized, and the powdered extracts were stored in light-protected tubes at −80 °C for further analysis. The extraction yield for the different solvents was calculated as a percentage of the initial mass of the plant, with the following yields: E:W 80% *v*/*v* = 17%, E:W 50% *v*/*v* = 13%, M:W 80% *v*/*v* = 20%, M:W 50% *v*/*v* = 16% and for the 100% aqueous extract = 9%.

### 4.3. Phenolic Compounds Identification and Quantification

Prior to the analyses, 100 mg of the extracts were mixed with 10 mL 80% *v*/*v* methanol, followed by sonication for 30 min (Branson 5510 equipment, Branson Ultrasonics, Brookfield, CT, USA), centrifugation at 1500× *g* for 10 min at 4 °C (Z326K, Hermle, Wehingen, Germany), and filtration using a 0.22 µm filter (Pall Corporation (Port Washington, NY, USA), GVS Porex, 25 mm). Total phenolic compounds (TPC) were determined through the Folin–Ciocalteu method [57], and the results were expressed in mg of gallic acid equivalents (GAE) per 100 g of lyophilized extract (LE). Total flavonoids (TF) were spectrophotometrically quantified by NaNO_2_ and AlCl_3_ oxidation with NaOH as reported by Zhishen et al. [58] and were indicated as mg of (+)-catechin equivalents (CE) per 100 g LE. Total condensed tannins (CT) were quantified using the method reported by Feregrino-Pérez et al. [59] and were also expressed as mg CE/100 g LE.

For the identification and quantification of specific phenolic compounds, a high-performance liquid chromatography (HPLC) method coupled with diode-array detection (DAD) was used [60]. Briefly, an HPLC Equipment (Series 1100, Agilent Technologies, Palo Alto, CA, USA) using a Zorbax XDB-C18 column (Agilent Technologies, 4.6 × 250 mm and 5 µm of granule size). The phenolic compound standards used for calibration were obtained from Sigma-Aldrich (St. Louis, MO, USA) and had a purity of ≥95% (Supplementary Table S1).

The column was thermostatically adjusted (35 ± 0.6 °C), and the flow rate was set at 1 mL/min. The mobile phase consisted of two solvents: 0.1% *v*/*v* acidified HPLC-grade water with acetic acid (A) and 100% HPLC-grade acetonitrile (B). A lineal gradient was set as follows: 80–83% A for 7 min, 83–60% A for 5 min, 60–50% A for 1 min, and 50–85% A for 2 min. Detection was carried out at 280 nm and 320 nm for hydroxycinnamic acids and flavonoids, respectively. A volume of 20 µL was injected. The quantification was conducted using HPLC-grade commercial standards of hydroxybenzoic acids (ellagic, 4-hydroxybenzoic, and gallic acids), hydroxycinnamic acids (sinapic, chlorogenic, caffeic, *p*-coumaric, and ferulic acids), 4-hydroxyphenylacetic acid, flavanols [(+)-catechin, (−)-epicatechin, and (−)-epigallocatechin-3-*O*-gallate], and flavonols (rutin and quercetin) A validation of the HPLC method for the quantification of phenolic compounds was performed, which included the construction of calibration curves, determination of the limits of detection (LOD) and quantification (LOQ), and evaluation of the reproducibility of the method. Specific parameters for the calibration curves, the limits of detection and quantification, as well as the reproducibility of retention times and chromatographic peak areas are presented in the Supplementary Material (Supplementary Table S2).

### 4.4. Antioxidant Capacity Determination

The antioxidant capacity was quantified by the inhibition of radicals. For this, 2,2-diphenyl-1-picrylhydrazyl (DPPH) [61], the ferric reducing antioxidant power (FRAP) [62], and the 2,2-azino-bis(3-ethylbenzothiazoline-6-sulfonic acid) (ABTS) [63] methods were used. The results were expressed in µM Trolox equivalents/g LE.

### 4.5. Cell Culture Assays

SW480 [SW-480] (ATCC CCL-228) human colon adenocarcinoma cells were acquired from the American Type Culture Collection (ATCC). The cells were seeded in 60 mm plates using Dulbecco’s Modified Eagle’s Medium (DMEM) (Gibco, Waltham, MA, USA) supplemented with 10% *v*/*v* fetal bovine serum (FBS) (Biowest, Lakewood Ranch, FL, USA) and 1% Antibiotic–Antimycotic solution (Gibco). The cells were maintained in a humidified CO_2_ (5%) atmosphere at 37 °C, where the medium was changed every 2 days until reaching an 80% confluence.

#### 4.5.1. Quantification of Cellular Metabolic Activity by 3-(4,5-Dimethylthiazol-2-yl)-2,5-Diphenyltetrazolium Bromide (MTT) Assay

The cells (1.5 × 10^4^ cells/well, 100 µL volume) were seeded in 96-well plates for 24 h. The cells were then exposed to the methanolic extract of the arils in serial dilutions (1, 5, 10, 15, 30, 50, and 100 µg/mL, dissolved in DMEM supplemented with 0.5% bovine seric albumin, BSA) for 24 h. After the incubation, DMEM was replaced with an MTT solution (0.5 mg/mL), previously prepared by dissolving the MTT in 0.22 µm-filtered and antibiotic and FBS-free DMEM (100 µL/well), and the cells were incubated for 24 h. Then, DMEM was removed, and dimethyl sulfoxide (DMSO) was added for 5 min. The absorbance was read at 562 nm in a spectrophotometer, and the half-lethal concentration (LC_50_) was calculated using a 4-parameter dose-response curve provided by GraphPad Prism v. 8.0 software (Dotmatics, Boston, MA, USA). Untreated cells were considered as the negative control. The results were expressed as metabolic activity against the negative control (%).

#### 4.5.2. Cell Necrosis Determination by Lactate Dehydrogenase (LDH) Assay

The cells (1.5 × 10^4^ cells/well, 100 µL volume) were cultivated as indicated in 3.5.1. for 24 h. Then, the cells were treated with 100 µL of the quantified *P. dulce* LC_50_ of the arils’ treatments from the MTT assay (prepared with DMEM + 0.5% BSA). The cell supernatants were then used to quantify LDH release using an LDH assay kit (K311-400, Biovision, Milpitas, CA, USA). The samples were read at 492 nm in a spectrophotometer and were expressed as cytotoxicity (%) using the following equation: [Abs_sample_ − Abs_negative control_/Abs_positive control_] × 100% − Abs_Negative control_, where Abs refers to the absorbance. Untreated cells were used as the negative control, and cells treated with Triton 100-X were used as the positive control. To determine a value for the positive control that represents all cells in the sample, a detergent (lysis solution: a 1:250 dilution of 9% w:v Triton X-100) is used to lyse the cells and allow staining of the entire population. To prepare positive control samples, 4 µL of lysis solution is added for every 100 µL of cells in the culture medium, and the samples are mixed using a plate shaker as described above. The samples are allowed to stand at room temperature, and the fluorescence is recorded. Note: The dynamic range of the plate reading fluorometer should be validated to ensure that it is capable of reading over the full fluorescence range for the chosen cell number and probe concentration [64].

#### 4.5.3. Apoptosis Quantification by Cell Cytometry

The cells (1 × 10^6^ cells/Petri dish plate) were cultured until reaching an 80% confluence, then treated with the *P. dulce* LC_50_ (3 mL/plate) prepared with DMEM + 0.5% BSA. Once trypsinized and collected by centrifugation (3000× *g* for 5 min), the cells were washed with phosphate-buffered saline (PBS) solution (1×) and adjusted to 1 × 10^6^ cells/mL. The Muse Dead Cells and Annexin V assay kit (MCH 100105, Millipore, Darmstadt, Germany) was used in a Guava Muse Cell Analyzer^®^ (Luminex, Austin, TX, USA). The results were expressed as the proportion of live, early apoptotic, late apoptotic, and total apoptotic cells. Untreated cells were used as the negative control.

#### 4.5.4. Cell Cycle Analysis by Flow Cytometry

The cells (3 × 10^5^ cells/60 mm Petri dish) were cultured until reaching an 80% confluence. The cells were exposed to the LC_50_ concentration of *P. dulce* treatments, prepared with DMEM + 0.5% BSA for 12 h, followed by trypsinization, centrifugation (3000× *g*, 5 min), PBS (1×) + 1 mM ethylenediaminetetraacetic (EDTA) washing, and ethanol (70% *v*/*v*) fixation for 4 h at −20 °C. Cell cycle analysis was performed using a Muse Cell Cycle Assay kit (MCH1006, Millipore) and analyzed through a Muse Cell Analyzer (Luminex). Untreated cells (DMEM + 0.05% BSA) were used as the negative control. The results were expressed as the percentage of total cells in each phase of the cell cycle: G0/G1, S, and G2/M.

#### 4.5.5. Assessment of p53 and KRAS Gene Expression by qPCR Analysis

The cells were cultured (3 × 10^5^ cells/60 mm Petri dish) and treated as previously indicated with the LC_50_ of *P. dulce*. Extraction and purification of mRNA were followed by adding 400 µL TRIzol reagent (Invitrogen, Carlsbad, CA, USA), and extracted RNA was resuspended in RNA and DNA-ase free water, which was used to quantify total RNA. Purity was assessed in a spectrophotometer (NanoDrop 2000/2000c, ThermoScientific, Waltham, MA, USA). Then, each 2 µg of mRNA was used for DNA_c_ synthesis using a kit (Maxima H Minus First Strand cDNA Synthesis, K1652, ThermoScientific). Primers for p53 and KRAS genes were designed considering their involvement in carcinogenesis and colorectal cancer apoptosis signaling (Supplementary Table S3). Gene sequences were analyzed in the UCSC Genome Browser of the University of California (https://genome.ucsc.edu/, accessed on 1 March 2024), and the primers were designed using the Primer3 website (https://primer3.ut.ee/, accessed on 1 March 2024), selecting a melting temperature (MT) of 60 ± 2 °C (for 20 ± 2 pb), 100–250 pb of final product size and GC ≥ 50%. Sequences were synthesized by Sigma-Aldrich Mexico (Toluca, Mexico). The qPCR reaction was performed in 96-well PCR plates using 3.4 µL of DNA-RNA-ase free water, 5 µL of SYBR^®^ Select Mater Mix for CFX (4472942, Applied Biosystems, Foster City, CA, USA), and 1 µL of cDNA for each reaction. A thermal cycler (CFX96, BioRead Lab. Inc., Hercules, CA, USA) was used with the following conditions: 95 °C for 10 min (15 s at 95 °C, 30 s at 60 °C, and 30 s at 72 °C) for 35 cycles. The analysis of the relative expression of the genes was reported using the 2^−ΔΔCt^ method, using GAPDH as the housekeeping gene.

### 4.6. Statistical Analysis

The results were expressed as the mean ± S.D. of three independent experiments in triplicate. After normality testing using Shapiro–Wilk’s test, normality plots, and homoscedasticity analyses, a one-way analysis of variance (ANOVA) was conducted, followed by a post hoc Tukey–Kramer’s test, where differences were established at *p* < 0.05. The statistical analysis was conducted using GraphPad Prism v. 8.0 software (Dotmatics, Boston, MA, USA). A Principal Components Analysis (PCA) was also conducted to assess the sample variability considering the combination of the drying methods and assayed solvents through FactoMineR (version 2.11) and FactoExtra R (version 1.0.7) software packages. A heatmap was also performed using the Pheatmap package in RStudio, and the concentrations of phenolic compounds obtained by HPLC-DAD were logarithmically transformed (log1p) to improve visibility in the graphical analysis.

## 5. Conclusions

The findings obtained in the present study demonstrate the significant impact that the drying method has on the phenolic profile and the antioxidant capacity of the aril of *P. dulce*. Oven drying combined with methanolic extraction (50% *v*/*v* M:W) provided a greater quantity of polyphenolic compounds. This is probably due to the release of the phenolic compounds found in the cell wall of the plant sample favored by the temperature at 40 °C, facilitating the extraction of these compounds. On the other hand, the freeze-drying method did not favor the same level of release. These results suggest that the oven-drying method optimizes the conservation and obtention of bioactive compounds and could be of considerable significance to the pharmaceutical and food industries. Furthermore, the methanolic extract of *P. dulce* promoted cell death by apoptosis in human colon adenocarcinoma cells (SW480), demonstrating cell cycle arrest in the G1 phase, suggesting that the polyphenolic compounds of *P. dulce* interfere with the DNA synthesis process, in the activation of Tp53 (tumor suppressor) and in the regulation of cell death by apoptosis. In addition, the reduction found in KRAS (an oncogene that is mutated in colon cancer) indicates an inhibition of the signaling pathways that favor cell survival and proliferation. However, in vivo studies are needed to evaluate the chemopreventive effect of the *P. dulce* aril on colon cancer.

## Figures and Tables

**Table 1:** Total phenolic compounds, total flavonoids, and total condensed tannins found in hydroalcoholic and aqueous extracts of *P. dulce* aril.

Extracts	TPC (mg GAE/100 g LE)		TF (mg CE/100 g LE)		CT (mg CE/100 g LE)	
	Oven-Drying	Freeze-Drying	Oven-Drying	Freeze-Drying	Oven-Drying	Freeze-Drying
Aqueous	892.50 ± 56.67 ^bA^	799.14 ± 33.94 ^aA^	5.85 ± 1.94 ^bA^	5.10 ± 2.40 ^aA^	0.18 ± 0.07 ^aA^	0.13 ± 0.03 ^aA^
80% *v*/*v* E:W	1061.01 ± 151.65 ^abA^	631.72 ± 28.85 ^bB^	20.93 ± 4.06 ^aA^	7.10 ± 4.31 ^aB^	0.16 ± 0.08 ^aA^	0.07 ± 0.01 ^bA^
50% *v*/*v* E:W	1038.99 ± 45.39 ^abA^	651.55 ± 36.33 ^bB^	17.11 ± 3.01 ^aA^	6.67 ± 1.79 ^aB^	0.19 ± 0.08 ^aA^	0.08 ± 0.01 ^bA^
80% M:W	1149.45 ± 69.27 ^aA^	637.36 ± 57.32 ^bB^	18.68 ± 2.52 ^aA^	7.90 ± 2.73 ^aB^	0.15 ± 0.07 ^aA^	0.08 ± 0.01 ^bA^
50% M:W	1094.50 ± 54.22 ^abA^	726.53 ± 34.06 ^abB^	16.84 ± 2.00 ^aA^	6.05 ± 1.99 ^aB^	0.17 ± 0.08 ^aA^	0.09 ± 0.02 ^abA^

Results are expressed as the mean ± SD of three independent experiments in triplicate. Different capital letters express significant differences (*p* < 0.05) (by Student’s *t*-test) between oven-dried and freeze-dried extracts (oven-dried vs. freeze-dried). Different lower-case letters express significant differences (*p* < 0.05) (by Tukey–Kramer test) between all extracts (different solvent types) for each drying method and determination. CE: (+)-Catechin equivalents; CT: Condensed tannins; E:W: Ethanol:water extract; GAE: Gallic acid equivalents; LE: Lyophilized extract; M:W: Methanol:water extract; TF: Total flavonoids; TPC: Total phenolic compounds.

**Table 2:** Antioxidant capacity of hydroalcoholic and aqueous extracts of *P. dulce* aril.

Extracts	DPPH ^1^		FRAP ^1^		ABTS ^1^	
	Oven-Drying	Freeze-Drying	Oven-Drying	Freeze-Drying	Oven-Drying	Freeze-Drying
Aqueous	37.82 ± 3.13 ^aA^	19.64 ± 0.87 ^bAB^	26.29 ± 1.97 ^bA^	35.03 ± 3.05 ^aA^	178.75 ± 9.63 ^aA^	151.84 ± 7.87 ^bA^
80% *v*/*v* E:W	43.03 ± 3.27 ^aA^	22.28 ± 0.97 ^bA^	33.12 ± 6.53 ^aA^	30.67 ± 1.33 ^aAB^	190.06 ± 16.27 ^aA^	159.43 ± 19.13 ^bA^
50% *v*/*v* E:W	42.43 ± 2.32 ^aA^	19.10 ± 0.83 ^bB^	30.71 ± 2.69 ^aA^	27.93 ± 2.06 ^aB^	200.26 ± 13.66 ^aA^	143.81 ± 11.87 ^bA^
80% M:W	43.67 ± 2.62 ^aA^	21.35 ± 1.30 ^bAB^	34.97 ± 1.56 ^aA^	29.66 ± 1.20 ^bB^	190.16 ± 5.59 ^aA^	153.15 ± 9.27 ^bA^
50% M:W	44.63 ± 2.00 ^aA^	21.44 ± 0.97 ^bAB^	31.74 ± 0.71 ^aA^	30.08 ± 0.81 ^aAB^	202.23 ± 6.32 ^aA^	158.34 ± 7.69 ^bA^

^1^ Values in µmol Trolox equivalents/g LE. The results are expressed as the mean ± S.D. of three independent experiments in triplicate. Results are expressed as the mean ± SD of three independent experiments in triplicate. Different capital letters express significant differences (*p* < 0.05) (by Student’s *t*-test) between oven-dried and freeze-dried extracts (oven-dried vs. freeze-dried). Different lower-case letters express significant differences (*p* < 0.05) (by Tukey–Kramer test) between all extracts (different solvent types) for each drying method and determination. ABTS: 2,2-Azino-bis(3-ethylbenzothiazoline-6-sulfonic acid); DPPH: 1,1-Diphenyl-2-picrylhydrazil assay; E:W: Ethanol:water extract; LE: Lyophilized extract; FRAP: Ferric reducing antioxidant power; M:W: Methanol:water extract.

**Table 3:** Content of individual phenolic acids detected in *P. dulce* aril using HPLC-DAD.

Extract	T	Hydroxybenzoic Acids ^1^			Hydroxycinnamic Acids ^1^					4-Hydroxyphenylacetic Acid ^1^	Total ^1^
		Ellagic Acid	4-Hydroxybenzoic Acid	Gallic Acid	Sinapic Acid	Chlorogenic Acid	Caffeic Acid	*p*-Coumaric Acid	Ferulic Acid		
Aqueous	O	ND	26.50 ± 1.53 ^fB^	922.30 ± 218.00 ^aA^	6.26 ± 0.41 ^bcA^	33.69 ± 0.71 ^cA^	2.99 ± 0.60 ^c^	19.84 ± 2.00 ^fB^	283.90 ± 0.18 ^bA^	193.00 ± 0.57 ^dB^	1488.48 ± 224.00 ^aA^
	FD	ND	77.63 ± 2.40 ^aA^	99.46 ± 4.93 ^bB^	6.20 ± 0.33 ^bcA^	11.85 ± 4.98 ^eB^	ND	300.00 ± 9.13 ^aA^	3.61 ± 0.03 ^cB^	289.30 ± 6.82 ^aA^	788.05 ± 28.63 ^bB^
80% *v*/*v* E:W	O	2.68 ± 0.02 ^b^	35.93 ± 4.43 ^eB^	762.90 ± 8.52 ^aA^	12.41 ± 3.04 ^aA^	49.69 ± 4.13 ^aA^	7.11 ± 0.04 ^a^	32.93 ± 0.23 ^efB^	307.90 ± 9.52 ^a^	202.90 ± 2.09 ^cdB^	1414.45 ± 32.01 ^aA^
	FD	ND	49.35 ± 2.46 ^bA^	32.29 ± 0.82 ^bB^	5.56 ± 0.12 ^bcB^	5.53 ± 0.12 ^fB^	ND	250.40 ± 3.21 ^bA^	ND	264.10 ± 3.66 ^bA^	607.23 ± 10.39 ^bcB^
50% *v*/*v* E:W	O	2.61 ± 0.05 ^b^	38.07 ± 0.45 ^deA^	818.20 ± 45.62 ^aA^	8.30 ± 0.29 ^bA^	36.72 ± 0.66 ^bcA^	4.62 ± 0.12 ^b^	32.80 ± 1.00 ^efB^	300.70 ± 4.08 ^a^	193.9 ± 2.36 ^dB^	1435.92 ± 54.63 ^aA^
	FD	ND	16.34 ± 1.58 ^gB^	35.67 ± 0.18 ^bB^	5.11 ± 0.24 ^cB^	13.03 ± 0.13 ^eB^	ND	195.20 ± 0.31 ^dA^	ND	210.00 ± 1.00 ^cA^	475.35 ± 3.43 ^cB^
80% M:W	O	2.59 ± 0.01 ^bB^	47.12 ± 6.00 ^bcdA^	867.90 ± 15.15 ^aA^	8.26 ± 0.53 ^bA^	42.52 ± 0.10 ^bA^	2.39 ± 0.03 ^cd^	45.59 ± 0.07 ^eB^	308.60 ± 2.91 ^a^	205.40 ± 0.72 ^cdB^	1530.37 ± 25.52 ^aA^
	FD	3.27 ± 0.19 ^aA^	47.53 ± 0.85 ^bcA^	38.21 ± 4.44 ^bB^	7.21 ± 0.56 ^bcA^	26.12 ± 0.40 ^dB^	ND	232.70 ± 8.52 ^cA^	ND	266.30 ± 8.40 ^bA^	621.34 ± 23.36 ^bcB^
50% M:W	O	2.53 ± 0.01 ^b^	45.08 ± 0.61 ^bcdB^	956.50 ± 5.18 ^aA^	7.75 ± 0.04 ^bcA^	49.30 ± 1.13 ^aA^	2.20 ± 0.05 ^d^	33.44 ± 0.81 ^dB^	311.60 ± 3.83 ^a^	211.10 ± 0.65 ^cA^	1619.50 ± 12.30 ^aA^
	FD	ND	40.91 ± 2.48 ^cdeA^	36.78 ± 2.40 ^bB^	4.91 ± 0.32 ^cB^	16.65 ± 0.99 ^eB^	ND	196.80 ± 10.63 ^efA^	ND	212.60 ± 10.84 ^cA^	508.65 ± 27.66 ^cB^

^1^ Values in mg/100 g LE. The results are expressed as the mean ± S.D. of three independent extractions in triplicate. Results are expressed as the mean ± SD of three independent experiments in triplicate. Different capital letters express significant differences (*p* < 0.05) (by Student’s *t*-test) between oven-dried and freeze-dried extracts (oven-dried vs. freeze-dried). Different lower-case letters express significant differences (*p* < 0.05) (by Tukey–Kramer test) between all extracts (different solvent types) for each drying method and determination. E:W: ethanol:water extract; FD: Freeze-drying; LE: Lyophilized extract; M:W: Methanol:water extract; ND: Not detected; O: Oven-drying; T: Treatment.

**Table 4:** Content of individual flavonoids detected in *P. dulce* aril using HPLC-DAD.

Extract	T	Flavanols ^1^			Flavonols ^1^		Total
		(+)-Catechin	(−)-Epicatechin	(−)-Epigallocatechin-3-*O*-gallate	Rutin	Quercetin	
Aqueous	O	13.87 ± 0.84 ^efA^	177.10 ± 0.58 ^dB^	15.74 ± 0.10 ^cdB^	31.08 ± 0.19 ^bA^	18.39 ± 0.01 ^bA^	256.18 ± 1.72 ^cB^
	FD	11.00 ± 0.33 ^fA^	205.80 ± 6.84 ^bcA^	18.80 ± 0.56 ^aA^	29.57 ± 1.21 ^abA^	16.49 ± 0.34 ^cB^	281.66 ± 9.27 ^bA^
80% *v*/*v* E:W	O	15.61 ± 0.17 ^eB^	203.30 ± 1.73 ^cA^	16.71 ± 0.18 ^bcA^	30.06 ± 8.76 ^b^	21.36 ± 0.08 ^aA^	287.04 ± 10.91 ^bA^
	FD	44.39 ± 0.42 ^bA^	23.32 ± 1.85 ^fB^	15.34 ± 0.20 ^dB^	ND	17.59 ± 0.68 ^bB^	100.64 ± 3.15 ^deB^
50% *v*/*v* E:W	O	15.77 ± 0.17 ^e^	199.00 ± 0.81 ^c^	15.85 ± 0.11 ^cd^	29.59 ± 0.68 ^b^	20.66 ± 0.13 ^a^	280.87 ± 1.91 ^bA^
	FD	32.72 ± 0.36 ^d^	28.99 ± 1.69 ^ef^	12.09 ± 0.13 ^f^	ND	12.46 ± 0.02 ^e^	86.26 ± 2.20 ^efB^
80% *v*/*v* M:W	O	15.54 ± 0.18 ^e^	210.90 ± 0.84 ^ab^	17.63 ± 0.05 ^b^	40.60 ± 0.43 ^a^	21.34 ± 0.03 ^a^	306.01 ± 1.53 ^aA^
	FD	55.42 ± 2.02 ^a^	31.39 ± 1.18 ^e^	13.90 ± 0.81 ^e^	ND	13.60 ± 0.49 ^d^	114.31 ± 4.50 ^dB^
50% *v*/*v* M:W	O	15.95 ± 0.17 ^eB^	213.20 ± 0.46 ^aA^	17.22 ± 0.10 ^bA^	40.68 ± 0.19 ^a^	21.47 ± 0.11 ^aA^	308.52 ± 1.04 ^aA^
	FD	36.54 ± 2.17 ^cA^	25.63 ± 1.67 ^efB^	10.44 ± 0.70 ^gB^	ND	12.66 ± 0.58 ^deB^	85.27 ± 5.12 ^fB^

^1^ Values in mg/100 g LE. The results are expressed as the mean ± S.D. of three independent extractions in triplicate. Different lower-case letters express significant differences (*p* < 0.05) (per Tukey-Kramer’s test) between all treatments. Different uppercase letters indicate significant differences (*p* < 0.05) (per Student’s *t*-test) between oven-drying and freeze-drying for each treatment. E:W: ethanol:water extract; FD: Freeze-drying; LE: Lyophilized extract; M:W: Methanol:water extract; ND: Not detected; O: Oven-drying; T: Treatment.

## Data Availability

Data will be available upon reasonable request.

## Supplementary Materials

The following supporting information can be downloaded at https://www.mdpi.com/article/10.3390/molecules30020233/s1, Table S1. Information on phenolic compound standards used for HPLC-DAD analysis, Table S2. Analytical parameters of phenolic compound standards used in HPLC-DAD analysis, Table S3. *Primer* sequences for the real-time polymerase chain reaction (qPCR), Table S4. Percentages and cumulative percentages of principal component analysis (PCA) of HPLC-DAD phenolic compounds from *P. dulce* aril extracts, Table S5. Total percentages and cumulative percentages of principal component analysis (PCA) of spectrophotometric components from *P. dulce* aril extracts, Figure S1. HPLC-DAD chromatograms of the phenolic compound extracts of *P. dulce*.
