# Supplementary material for: Effect of Drying Methods on the Phenolic Profile and Antioxidant Capacity of Pithecellobium dulce (Roxb.) Benth. Aril and Its Inhibitory Properties on Human SW480 Colon Adenocarcinoma Cells
Source: Molecules. 2025 Jan 9;30(2):233. doi: 10.3390/molecules30020233 (PMC11767361; doi:10.3390/molecules30020233)
Supplement: Supplementary file 1 [file molecules-30-00233-s001.zip › molecules-3270583-supplementary.pdf]

Table S1. Phenolic compound standards used for HPLC-DAD analysis

Standard name	Molecular Weight (g/mol)	Product Reference	Purity Grade
Phenolic acids			
Ellagic acid	302.19	E2250	≥ 95.0 %
4-hydroxybenzoic acid	138.12	240141	≥ 99.0 %
Gallic acid	188.13	27645	≥ 98.50 %
Chlorogenic acid	354.31	C3878	≥ 95.0 %
Caffeic acid	180.16	C0625	≥ 98.0 %
p-Coumaric acid	164.16	C9008	≥ 98.0 %
Ferulic acid	194.18	W518301	≥ 99.0 %
4-hydroxyphenylacetic acid	152.15	H50004	≥ 98.0 %
Flavonoids			
(+)-Catechin	290.27	C1251	≥ 98.0 %
(-)-Epicatechin	290.27	E4018	≥ 98.0 %
(-)-Epigallocatechin-3-*O*-gallate	458.37	E4143	≥ 95 %
Rutin	610.52	R5143	≥ 94 %
Quercetin	302.24	Q4951	≥ 95 %

Table S2. Analytical parameters of phenolic compound standards used in HPLC-DAD analysis

Standard name	Detection wavelength (nm)	RT (min)	Linearity range (mg/mL)	Regression coefficient (R^2)	Calculated Equation	LOD (mg/mL)	LOQ (mg/mL)
Phenolic acids							
Ellagic acid	280	3.34	0-100	0.982	y: 10.950x + 2.591	0.01	0.56
4-hydroxybenzoic acid	280	12.34	0-100	0.990	y: 2.441x + 0.1016	0.22	0.98
Gallic acid	280	7.49	0-100	0.999	y: 29.814x + 125.69	0.16	0.52
Chlorogenic acid	280	10.57	0-100	0.999	y: 56.690x - 208.28	0.11	0.38
Caffeic acid	280	11.82	0-100	0.999	y: 75.693x + 382.11	0.11	0.38
p-Coumaric acid	280	14.42	0-100	0.999	y: 129.146x + 60.01	0.61	2.04
Ferulic acid	280	15.34	0-100	0.999	y: 98.987x - 621.23	0.31	1.03
4-hydroxyphenylacetic acid	280	9.88	0-100	0.999	y: 5.3642x - 5.7511	0.34	1.10
Flavonoids							
(+)-Catechin	320	10.85	0-100	0.998	y:12.77x + 11.607	0.05	0.17
(-)-Epicatechin	320	11.55	0-100	0.991	y: 7.434x + 16.403	0.03	0.22
(-)-Epigallocatechin-3-*O*-gallate	320	11.79	0-100	0.999	y: 12.14x – 1.7373	0.12	0.41
Rutin	320	12.89	0-100	0.999	y: 18.14x - 35.55	0.04	0.14
Quercetin	320	18.44	0-100	0.998	y: 45.26x - 565.14	0.08	0.27

Table S3. *Primer* sequences for the quantitative real-time polymerase chain reaction (qPCR)

Gene	Forward (5′-3′)	Reverse (3′-5′)
p53	CCAACAACACCAGCTCCTCT	TCAGGAAGTAACACCATCGTAAG
Kras	TGTGATTTGCCTTCTAGAACAGT	ACACCCTGTCTTGTCTTTGCT

Table S4. Total percentage and cumulative percentage of PCA analysis of HPLC-DAD identified phenolic compounds.

Principal component (PCA)	Percentage	Cumulative Percentage
1	64.22	64.22
2	18.65	82.87
3	9.83	92.70
4	3.58	96.27
5	1.94	98.22
6	0.98	99.19
7	0.46	99.65
8	0.29	99.94
9	0.06	100.00
10	0.00	100.00

Table S5. Total percentage and cumulative percentage of PCA analysis of spectrophotometric parameters quantified from *P. dulce* extracts.

Principal component (PCA)	Percentage	Cumulative Percentage
1	76.561	76.561
2	16.406	92.967
3	5.287	98.254
4	0.942	99.196
5	0.638	99.834
6	0.166	100.00

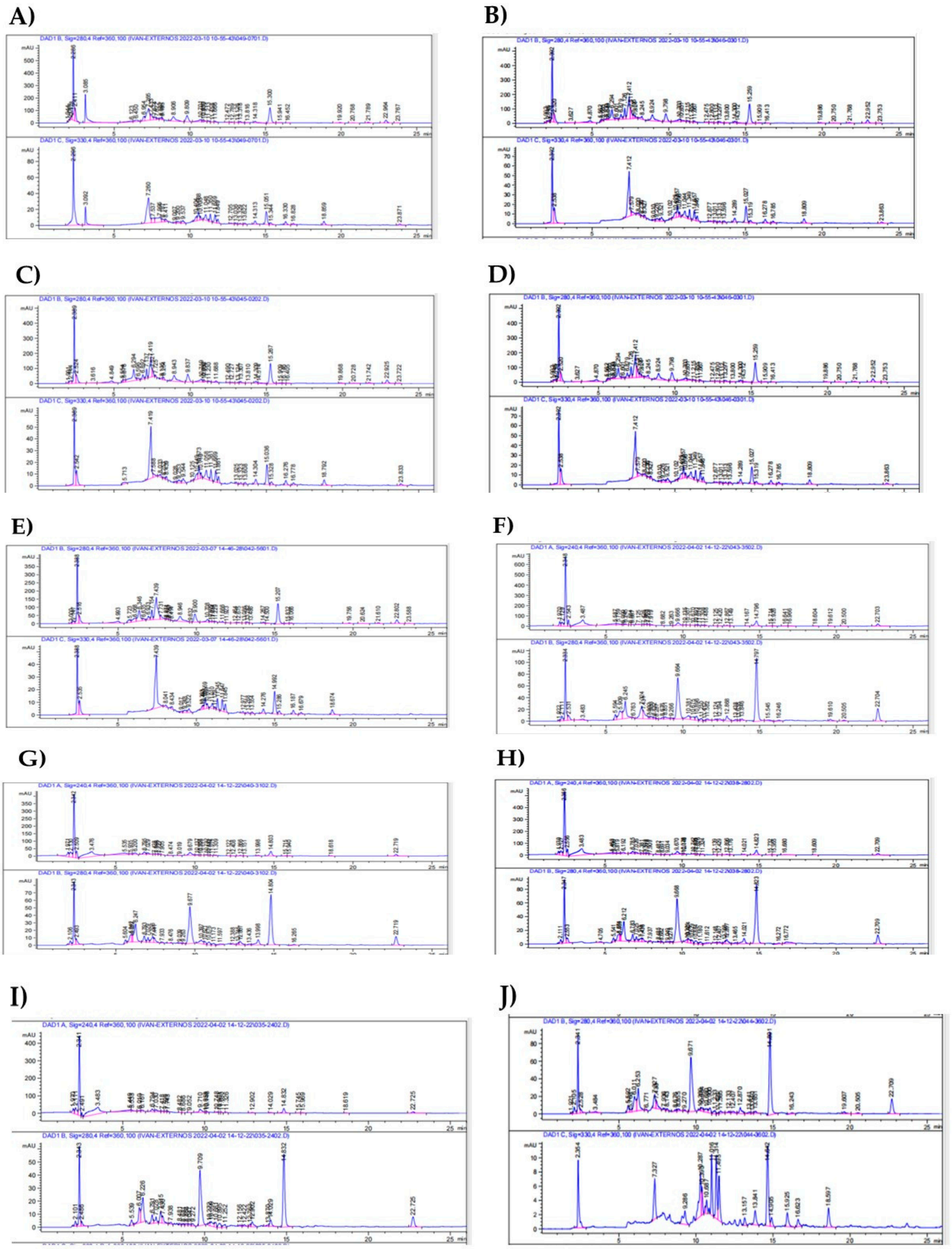

Figure S1. HPLC-DAD chromatograms of the phenolic compound extracts of *P. dulce*. **A-E) Oven dried extracts:** A) methanol-water 80:20 v/v, B) methanol-water 50:50 v/v, C) ethanol-water 80:20 v/v, D) ethanol-water 50:50 v/v, E) water 100%. **F-J) Freeze dried extracts:** F) methanol-water 80:20 v/v, G) methanol-water 50:50 v/v, H) ethanol-water 80:20 v/v, I) ethanol-water 50:50 v/v, J) 100% water.
